# Effect of catheter needle caliber on polidocanol foam stability in foam sclerotherapy

**DOI:** 10.3389/fneur.2024.1417788

**Published:** 2024-05-22

**Authors:** Sajjad Azmoun, Yiran Liu, Medina Tursun, Shaohua Liu

**Affiliations:** ^1^Department of Plastic, Cosmetic and Burn Surgery, Qilu Hospital of Shandong University, Jinan, China; ^2^Department of Endocrinology, Qilu Hospital of Shandong University, Cheeloo College of Medicine, Shandong University, Jinan, China; ^3^Department of Oral and Maxillofacial Surgery, Qilu Hospital of Shandong University, Jinan, China

**Keywords:** needle caliber, catheter needle, syringe needle, polidocanol, hyaluronic acid, foam stability, Tessari method

## Abstract

**Background:**

Although sclerotherapy is widely used to treat vascular malformations (VMs), it is associated with several challenges. One significant issue is the insufficient understanding of the influence of various factors on the stability of polidocanol (POL) foam used in sclerotherapy.

**Objective:**

This study aimed to explore the effect of the catheter needle caliber on foam stability when using POL with or without hyaluronic acid (HA) for the treatment of VMs.

**Methods and materials:**

The Tessari method generated sclerosant foam using POL both with and without HA. We used catheters and syringe needles of various calibers, and the resulting foam was transferred into new syringes to facilitate a comparison of foam stability. Foam half-life (FHT) was utilized as a metric to assess foam stability.

**Results:**

The study found that narrower needle calibers produced a more stable foam when POL was used alone; however, no significant effect was observed when HA was added. Furthermore, when the foam was expelled using catheters and syringe needles of the same size, no noticeable changes in the stability were observed.

**Conclusion:**

When choosing needles of varying calibers, their effect on foam stability should be carefully considered, particularly when the foam contains HA.

## 1 Introduction

Foam sclerotherapy is commonly used to treat vascular malformations (VMs), especially large VMs of the head and neck area (1–3). Foam sclerotherapy is a safe and effective therapeutic option for hemorrhoids (4). In foam sclerotherapy, achieving a stable sclerosant foam for optimal results is crucial because poor foam stability can potentially result in serious complications (5, 6). Blisters, paradoxical embolism, orbital compartment syndrome, and transient weakness of the facial nerve branch are complications that can arise when treating head and neck VMs (7).

Recently, the management of vascular anomalies affecting the maxillofacial, rectal, and gastroesophageal regions demonstrated foam’s efficacy, underscoring this therapeutic technique’s versatility (8).

Various factors influence the quality and stability of sclerosant foam, including the incorporation of surface-active agents (9), liquid-to-air ratio (10), type and concentration of sclerosant (11), use of different gases (12), and the pushing rate in the foam-making procedure (13).

Hyaluronic acid (HA) is a large, non-sulfated glycosaminoglycan that is the main component of the extracellular matrix (14). The addition of small amounts of HA has been demonstrated to significantly improve the overall stability of foams (15) while ensuring their safe and effective utilization in the treatment of VMs (3).

Once the sclerosant foam is prepared, the foam should be promptly injected into the veins affected by VMs, either through a syringe needle or with a catheter needle, which is mostly used for treating large VMs (16). It has been observed that foam stability varies depending on the caliber of the needle through which it is injected (6). However, the influence of the needle catheter caliber on foam stability remains unclear. This study aimed to investigate the impact of needle catheter caliber on foam stability using polidocanol (POL), a commonly used sclerosing agent, in combination with HA.

## 2 Materials and methods

We tested the stability of polidocanol sclerosant foam prepared with a liquid-to-air ratio of 1:4 using the Tessari technique. The experiment used a 1% solution of POL (SHAANXI TIANYU Pharmaceutical Co., Ltd.) and two 10 mL syringes (SHANDONG XINHUA ANDE Medical Supplies Co., Ltd.) with a three-way stopcock (Discofix). Eight distinct groups were developed and evenly dispersed across the two experiments. Each group used a disposable syringe with a 0.70 mm needle size and three catheter needles of various calibers. Hyaluronic acid (SHANDONG BOSHILUN FURUIDA Pharmaceutical Co., Ltd.) was used as an enhancer to improve foam stability.

### 2.1 Catheter needle selection

**Figure 1:**
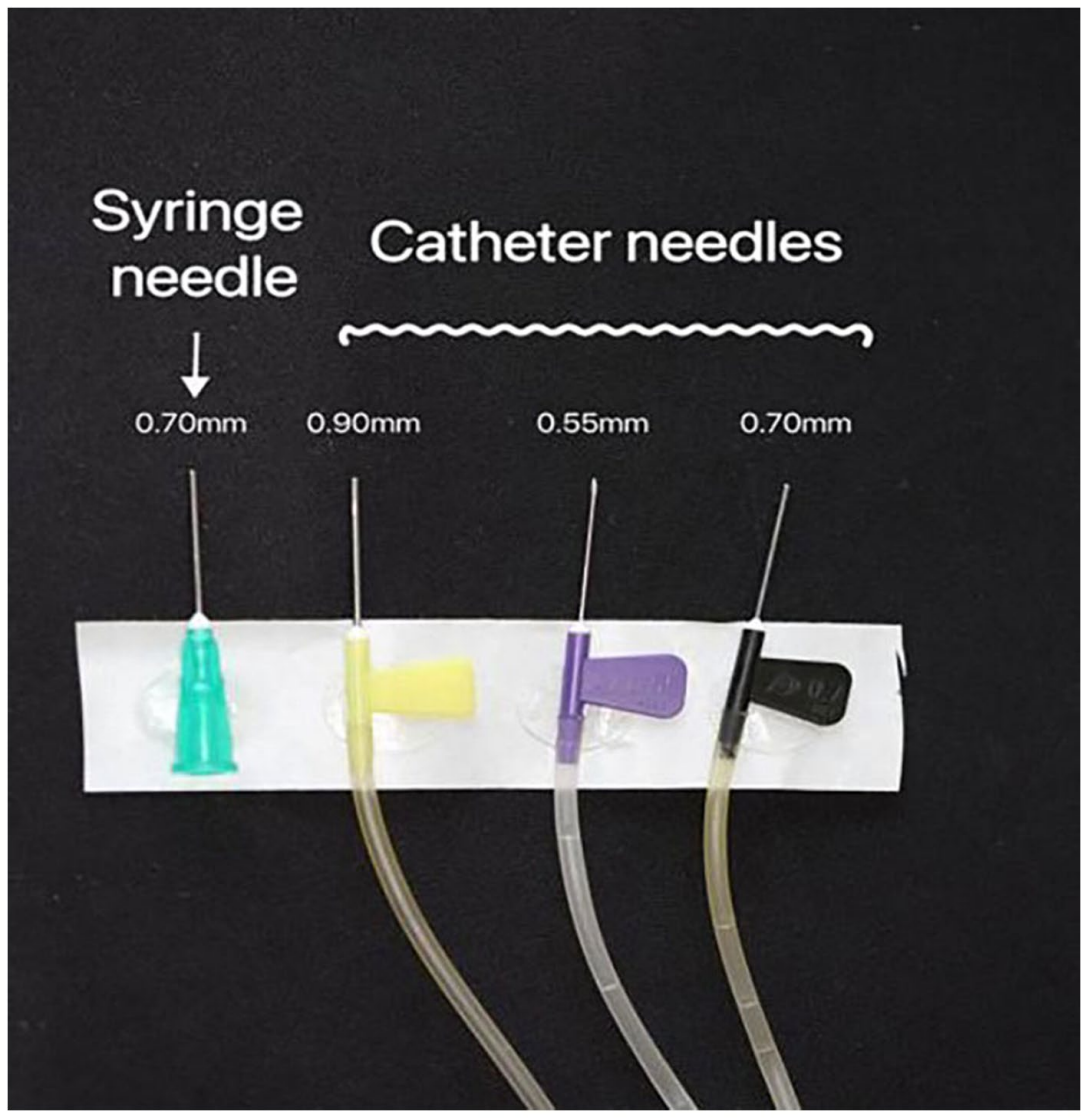
The needle catheters (0.55 mm, 0.70 mm, and 0.90 mm) and the 0.70 mm caliber disposable syringe needle were used. All the needles were trimmed to the same length.

Three catheter needles (WEIHAI WEIGAO Blood Collection Supplies Co., Ltd.) with diameters ranging from largest to narrowest (0.90 mm, 0.70 mm, and 0.55 mm) were employed. A disposable syringe with a 0.70 mm needle caliber (SHANDONG XINHUA ANDE Medical Supplies Co., Ltd.) was used in our experimental setup. To standardize the length of the catheter needle, we cut 0.90 mm, 0.70 mm, and 0.70 mm disposable syringe needles to match the length of the 0.55 mm catheter needle using a cutting machine. This ensured that all the needles used in the study were identical in length (Figure 1).

#### 2.1.1 Experiment 1: needle caliber and POL foam-making

**Figure 2:**
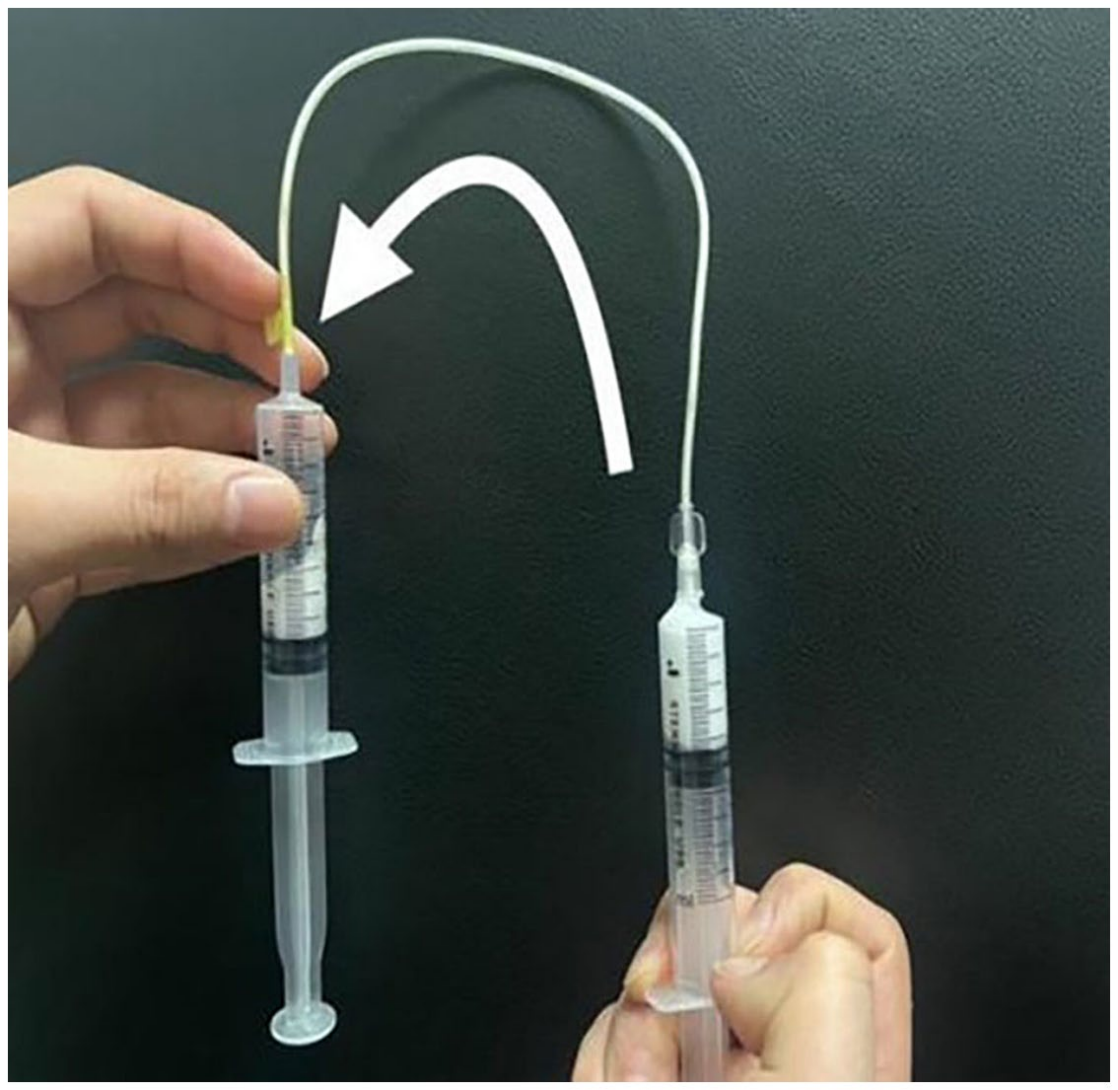
The POL sclerosant foam was transferred through a catheter needle to a new syringe.

Two syringes were connected to a three-way tap, with one syringe loaded with 2 mL of the POL solution and the other syringe filled with 8 mL of room air. The Tessari method was employed to produce the foam, which involved 20 cycles of pushing back and forth (13, 17). The resulting foam-containing syringe was promptly removed from the three-way tape. Using catheter needles 0.55 mm, 0.70 mm, and 0.90 mm in diameter and a syringe needle 0.70 mm in diameter, the sclerosant foam was gently transferred to an empty syringe for observation (Figure 2). The transfer time of the foam was meticulously controlled at 10 s for each needle caliber.

The foam half-life (FHT) was recorded to determine its stability. The rest of the plunger’s thumb was set on the table, the syringe was checked to ensure that it was steady and upright, the stopwatch was started immediately, and the drainage liquid extraction of the lower foam section was closely monitored. The FHT was recorded when 1 mL liquid emerged from the entire foam.

#### 2.1.2 Experiment 2: needle caliber and POL + HA foam-making

For this experiment, the method from Experiment 1 was slightly modified. HA (0.05 mL) was added to 2 mL of the POL solution loaded into one syringe, whereas the other syringe was filled with 8 mL of room air. The syringes were connected to three-way tape. Apart from the addition of HA, all other aspects of the experimental procedure, including the FHT detection, were consistent between the two experiments.

Each test was conducted 4 times by the same operator to ensure consistency and reliability in the experimental process. Fresh equipment was used for each repetition to ensure the generation of pristine foam. The room temperature was maintained at 25°C to ensure homogeneity across the experiments.

ANOVA was conducted to detect any difference in FHT, where *p* < 0.05 was regarded as a significant difference.

## 3 Results

### 3.1 Experiment 1

Among the needle catheters, those with diameters of 0.90 mm and 0.70 mm, as well as a disposable syringe with a 0.70 mm needle size, showed similar FHT values. Conversely, needles with a diameter of 0.55 mm exhibited the longest FHT duration, indicating superior stability of the sclerosant foam. Table 1 shows how the size of the needle affected the stability of the POL foam across the four groups.

**Table 1:** FHTs of different needle catheter calibers in POL foam (*n* = 4).

Needle caliber (mm)	FHT (s)	FHT (s)	FHT (s)	FHT (s)	FHT (s) (mean ± SD)
0.90 catheter	114	118	116	120	117 ± 2.6
0.70 catheter	115	116	116	121	117 ± 2.7
0.55 catheter	145	139	136	134	139 ± 4.8
0.70 syringe	110	119	118	119	117 ± 4.4

**Figure 3:**
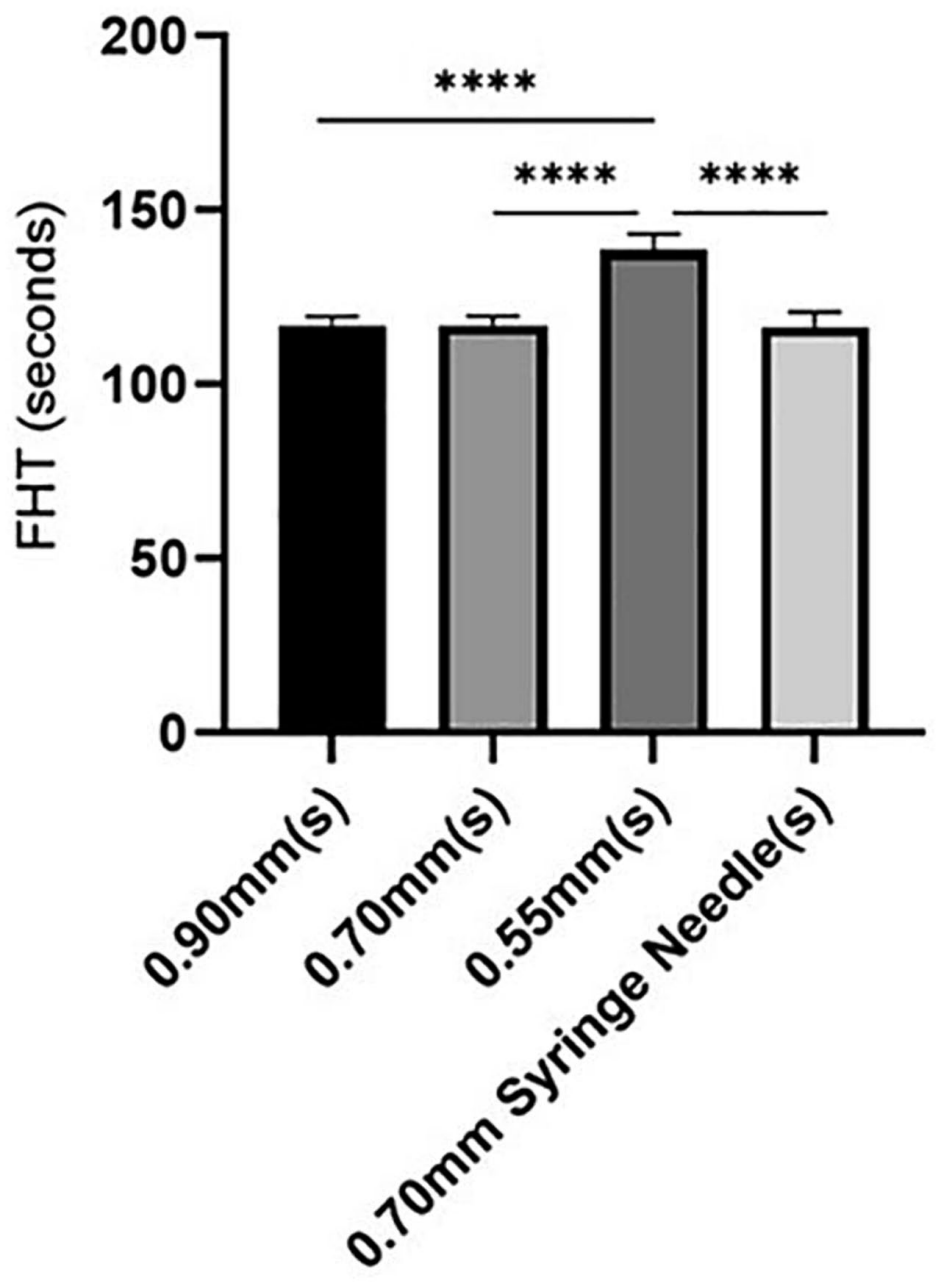
FHT in POL foam through different catheter needles and 0.70 mm syringe needle.

In the POL solution, a significant difference (*p* < 0.0001) was observed in the 0.55 mm group compared to the other groups. In contrast, no significant differences were detected among the remaining three groups (Figure 3).

### 3.2 Experiment 2

**Figure 4:**
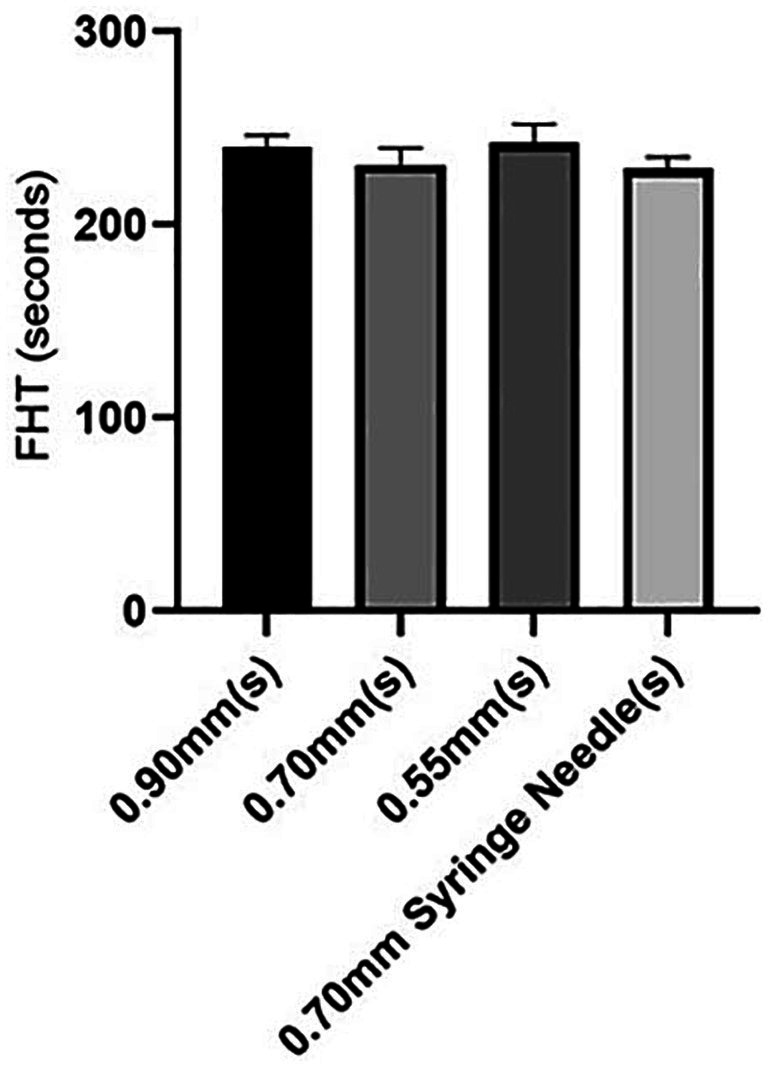
FHT in POL + HA foam through different catheter needles and 0.70 mm syringe needle.

The impact of needle size on foam stability in the presence of HA added to POL was investigated, as outlined in Table 2. Surprisingly, our results revealed that changing the needle caliber did not significantly influence the stability of the foam produced using POL combined with HA. Statistical analysis revealed no notable differences among the foams injected through needles of 0.55 mm, 0.70 mm, or 0.90 mm and those injected through a disposable syringe with a 0.70 mm needle caliber (*p* = 0.075) (Figure 4).

**Table 2:** FHTs of different needle catheter calibers in POL + HA foam (*n* = 4).

Needle caliber (mm)	FHT (s)	FHT (s)	FHT (s)	FHT (s)	FHT (s) (mean ± SD)
0.90 catheter	249	239	235	239	240 ± 6.0
0.70 catheter	237	233	218	236	231 ± 8.8
0.55 catheter	246	253	241	230	242 ± 9.7
0.70 syringe	229	228	237	222	229 ± 6.2

Remarkably, both the 0.70 mm catheter needle and disposable syringe with a 0.70 mm needle caliber showed identical FHT.

## 4 Discussion

Our findings highlight the significance of needle caliber in affecting foam stability among various factors. Specifically, when utilizing only POL sclerosant foam, we observed a shorter FHT with larger catheter needles and a 0.70 mm syringe needle, suggesting the potential for quicker dispersion and breakdown of the foam. Conversely, employing a narrower-caliber catheter needle resulted in enhanced stability.

In the second experiment, which involved POL + HA sclerosing foam, we did not observe a correlation between the needle caliber and foam stability. This finding indicates that the effect of needle caliber on foam stability varies between foam formulations with and without HA. The addition of HA significantly enhanced foam stability, thereby diminishing the influence of needle caliber on foam properties. Although the exact reason for this observation remains uncertain, we speculate that any potential effect of the needle caliber on the stability of the HA-containing foam may be too subtle to discern.

According to Burdick et al. (18), HA can be chemically altered to create various physical forms, including hydrogels, that are appropriate for preclinical and clinical use. Furthermore, the combination of POL and HA using the Tessari method for foam sclerotherapy has yielded a more stable and effective foam solution for treating VMs (3, 19).

Skuła et al. (6) conducted a commendable study on the influence of needle caliber on foam stability, shedding light on the significant impact of needle caliber and length. A meticulous examination revealed that both the needle caliber and length play a role in determining foam stability. However, given the nascent stage of this research area, a plethora of avenues for further exploration remain. Throughout our experiments, we used uniformly sized needles to validate the findings. Additionally, three different sizes of catheter needle calibers were used alongside only one syringe needle, a combination that has not been previously explored in similar studies. This distinctive approach adds depth to our understanding and underscores the importance of expanding research in this field.

We also discovered that foam stability was equivalent when using either a 0.70 mm direct needle or a 0.70 mm catheter needle. Despite the unknown reasons behind this, the choice between needle types, whether a syringe needle or catheter needle, may not be influenced by their impact on stability.

There are several foam production procedures in foam sclerotherapy, such as the double-syringe system (DSS) marketed as an Easyfoam kit and the ultrasonic approach (8). Varixio is another method of creating foam that uses an automated device to inject the foam (4). The Tessari technique and the DSS are widely employed methods for generating foam sclerosants (20) because they share similar mechanisms. In this study, the Tessari method was used to create foam. If DSS had been used, we would have expected the results to be comparable.

It is essential to emphasize that foam stability is influenced by various factors, and the needle caliber is only one of them. A limitation of our study was the restricted variety of catheter needles used, with only three different sizes available from the same manufacturer. To enhance the comprehensiveness of future studies, a broader range of needle calibers from diverse manufacturers should be considered. Another limitation was the use of only POL. Future studies should incorporate other sclerosant agents at varying concentrations to provide a more comprehensive understanding of foam stability in different formulations. Furthermore, the foam preparation using the Tessari method is manual and susceptible to multiple factors. Each experiment was repeated four times. Although the results of each independent experiment were similar, conducting more repetitions may have yielded more precise results.

## 5 Conclusion

Our study revealed that the needle caliber had an impact on the stability of the foam produced with POL alone to some extent. However, when POL was combined with HA, the caliber of the needle did not significantly affect foam stability. It is suggested that when selecting needles of different calibers, their influence on foam stability should be considered, depending on whether the foam contains HA.

## Data availability statement

The raw data supporting the conclusions of this article will be made available by the authors, without undue reservation.

## Author contributions

SA: Writing – original draft. YL: Writing – original draft. MT: Writing – original draft. SL: Writing – review & editing.
